# Supplementary material for: Attention periodically samples competing stimuli during binocular rivalry
Source: eLife. 2018 Dec 3;7:e40868. doi: 10.7554/eLife.40868 (PMC6298779; doi:10.7554/eLife.40868)
Supplement: Transparent reporting form [file elife-40868-transrepform.docx]

***eLife’s* transparent reporting form**

We encourage authors to provide detailed information *within their submission* to facilitate the interpretation and replication of experiments. Authors can upload supporting documentation to indicate the use of appropriate reporting guidelines for health-related research (see [EQUATOR Network](http://www.equator-network.org/%20)), life science research (see the [BioSharing Information Resource](https://biosharing.org/)), or the [ARRIVE guidelines](http://www.plosbiology.org/article/info:doi/10.1371/journal.pbio.1000412) for reporting work involving animal research. Where applicable, authors should refer to any relevant reporting standards documents in this form.

If you have any questions, please consult our Journal Policies and/or contact us: [editorial@elifesciences.org](mailto:editorial@elifesciences.org).

**Sample-size estimation**

- You should state whether an appropriate sample size was computed when the study was being designed
- You should state the statistical method of sample size computation and any required assumptions
- If no explicit power analysis was used, you should describe how you decided what sample (replicate) size (number) to use

Please outline where this information can be found within the submission (e.g., sections or figure legends), or explain why this information doesn’t apply to your submission:

- No explicit power analyses were performed.
- Our investigation was designed for electroencephalographic (EEG) analysis as an extension of a previous psychophysical experiment, which had 9 participants (Lunghi et al., Journal of Neuroscience, 2014).
- Previous behavioural investigations of attentional sampling have tested between 14-16 participants (e.g. Landau & Fries, 2012; Fiebelkorn et al., 2013), and previous neurophysiological studies test between 15 (e.g. Busch & VanRullen, 2010) and 22 participants (e.g. Landau et al., 2015).
- To ensure a robust estimation of both behavioural and neurophysiological effects, we recruited a sample of 35 participants, 34 of whom each contributed approximately 4 hours of data each to this study.

**Replicates**

- You should report how often each experiment was performed
- You should include a definition of biological versus technical replication
- The data obtained should be provided and sufficient information should be provided to indicate the number of independent biological and/or technical replicates
- If you encountered any outliers, you should describe how these were handled
- Criteria for exclusion/inclusion of data should be clearly stated
- High-throughput sequence data should be uploaded before submission, with a private link for reviewers provided (these are available from both GEO and ArrayExpress)

Please outline where this information can be found within the submission (e.g., sections or figure legends), or explain why this information doesn’t apply to your submission:

- This experiment was performed once.
- No biological or technical replicates.
- Each participant contributed approximately 4 hours of combined EEG and behavioural data, split over two days. Inclusion criteria were normal or corrected to normal vision. Exclusion criteria included failure to attend both days of testing.
- Outliers were identified on the basis of failure to follow task instructions and excessive movement during EEG recording (N=1).

**Statistical reporting**

- Statistical analysis methods should be described and justified
- Raw data should be presented in figures whenever informative to do so (typically when N per group is less than 10)
- For each experiment, you should identify the statistical tests used, exact values of N, definitions of center, methods of multiple test correction, and dispersion and precision measures (e.g., mean, median, SD, SEM, confidence intervals; and, for the major substantive results, a measure of effect size (e.g., Pearson’s r, Cohen’s d)
- Report exact p-values wherever possible alongside the summary statistics and 95% confidence intervals. These should be reported for all key questions and not only when the p-value is less than 0.05.

Please outline where this information can be found within the submission (e.g., sections or figure legends), or explain why this information doesn’t apply to your submission:

- All statistical analysis methods are detailed thoroughly in the manuscript figure legends, Methods (p30-42), as well as supplementary figures.
- For probability over time of perceiving the congruent flicker (Figure 2a, c, d), significant differences between cue types were determined using repeated-measures ANOVA followed by planned comparisons (comparing probability for attended/unattended, low Hz / high Hz/ and visual cue combinations). We tested for differences at each time point (16.7 ms steps, over 7 seconds), and use FDR q = .05 as the statistical threshold when correcting for multiple comparisons across time points.
- To compare Mismatch and Match CDF (Figure 3b), we applied a similar analysis (running paired samples t-tests), testing each 16.7ms (over 10 seconds) and correcting for multiple comparisons with FDR q = 05.
- To test the spectral amplitude of first switches (Figure 3f), we employed a two-stage statistical significance test based on non-parametric permutations, well established for the analysis of electrophysiological data (Maris & Oostenveld, 2007). In the first stage, a minimum of two adjacent significant frequencies (at p<.005 uncorrected) were first identified through a non-parametric randomization procedure. At the second stage, we compared clustered amplitudes (from the first stage) to the maximum clustered level amplitudes observed from a null-distribution of surrogate data, and retained our observed clusters (from the first stage) as significant when exceeding the top 95% of this null distribution (or p_cluster_ < .05). Extra details are documented in the *Methods* / *Spectral analysis of first switches* (p37-38) and the permutation null-distributions used to calculate statistical significance displayed in Figure 3 supplement 2.
- A similar two-stage procedure was performed to test the spatial topography of ITPC. Here a minimum of two neighbouring electrodes (maximum inter-electrode distance = 3.5 cm) which satisfied the first stage of analysis (p<.05 uncorrected) were retained for the second stage. Clustered test-statistics were then compared to the maximum clustered test-statistics generated by shuffling condition labels in our null distribution. The observed cluster-level t-scores were regarded as significant when exceeding the top 95% of this null distribution. Additional information on this process is detailed on in *Methods* / *ITPC statistics* on pages 40-41, with the null distributions used to calculate significance presented in Figure 4 supplement 1, and Figure 5 supplement 1.
- Exact p-values are reported throughout the manuscript where possible. When clustered test-statistics exceeded 100% of the permutation null distribution, they were reported as p_cluster_ < .001.

(For large datasets, or papers with a very large number of statistical tests, you may upload a single table file with tests, Ns, etc., with reference to sections in the manuscript.)

**Group allocation**

- Indicate how samples were allocated into experimental groups (in the case of clinical studies, please specify allocation to treatment method); if randomization was used, please also state if restricted randomization was applied
- Indicate if masking was used during group allocation, data collection and/or data analysis

Please outline where this information can be found within the submission (e.g., sections or figure legends), or explain why this information doesn’t apply to your submission:

- On one of their two testing days, participants were instructed to ignore crossmodal cues. Group allocation was randomized for the day of this condition (day 1 for n=18, day 2 for n=16).
- No other experimental groups were formed.

**Additional data files (“source data”)**

- We encourage you to upload relevant additional data files, such as numerical data that are represented as a graph in a figure, or as a summary table
- Where provided, these should be in the most useful format, and they can be uploaded as “Source data” files linked to a main figure or table
- Include model definition files including the full list of parameters used
- Include code used for data analysis (e.g., R, MatLab)
- Avoid stating that data files are “available upon request”

Please indicate the figures or tables for which source data files have been provided:

- Figure 3 supplement 2, Figure 4 supplement 1, and Figure 5 supplement 1 display the null distributions used to calculate spectral and spatial clustered significance.
- Matlab code used for this experiment and data analysis will be made publicly available at github.com/Davidson-MJ
- Data for this experiment will be publicly available on the Monash University figshare account (monash.figshare.com).
